# *Pseudomonas aeruginosa *toxin ExoU induces a PAF-dependent impairment of alveolar fibrin turnover secondary to enhanced activation of coagulation and increased expression of plasminogen activator inhibitor-1 in the course of mice pneumosepsis

**DOI:** 10.1186/1465-9921-12-104

**Published:** 2011-08-05

**Authors:** Gloria-Beatriz Machado, Albanita V de Oliveira, Alessandra M Saliba, Carolina D Mallet de Lima, José HR Suassuna, Maria-Cristina Plotkowski

**Affiliations:** 1 Department of Microbiology, Immunology and Parasitology, Medical Sciences Faculty, State University of Rio de Janeiro, 20 551-030, Rio de Janeiro, Brazil; 2 Department of Patology and Laboratories, Medical Sciences Faculty, State University of Rio de Janeiro, 20 551-030, Rio de Janeiro, Brazil; 3 Department of Internal Medicine, Medical Sciences Faculty, State University of Rio de Janeiro, 20 551-030, Rio de Janeiro, Brazil

**Keywords:** Acute respiratory distress syndrome, ExoU, platelet activating factor (PAF), plasminogen activator inhibitor type I (PAI-1), *Pseudomonas aeruginosa*, sepsis

## Abstract

**Background:**

ExoU, a *Pseudomonas aeruginosa *cytotoxin with phospholipase A_2 _activity, was shown to induce vascular hyperpermeability and thrombus formation in a murine model of pneumosepsis. In this study, we investigated the toxin ability to induce alterations in pulmonary fibrinolysis and the contribution of the platelet activating factor (PAF) in the ExoU-induced overexpression of plasminogen activator inhibitor-1 (PAI-1).

**Methods:**

Mice were intratracheally instilled with the ExoU producing PA103 *P. aeruginosa *or its mutant with deletion of the *exoU *gene. After 24 h, animal bronchoalveolar lavage fluids (BALF) were analyzed and lung sections were submitted to fibrin and PAI-1 immunohistochemical localization. Supernatants from A549 airway epithelial cells and THP-1 macrophage cultures infected with both bacterial strains were also analyzed at 24 h post-infection.

**Results:**

In PA103-infected mice, but not in control animals or in mice infected with the bacterial mutant, extensive fibrin deposition was detected in lung parenchyma and microvasculature whereas mice BALF exhibited elevated tissue factor-dependent procoagulant activity and PAI-1 concentration. ExoU-triggered PAI-1 overexpression was confirmed by immunohistochemistry. In *in vitro *assays, PA103-infected A549 cells exhibited overexpression of PAI-1 mRNA. Increased concentration of PAI-1 protein was detected in both A549 and THP-1 culture supernatants. Mice treatment with a PAF antagonist prior to PA103 infection reduced significantly PAI-1 concentrations in mice BALF. Similarly, A549 cell treatment with an antibody against PAF receptor significantly reduced PAI-1 mRNA expression and PAI-1 concentrations in cell supernatants, respectively.

**Conclusion:**

ExoU was shown to induce disturbed fibrin turnover, secondary to enhanced procoagulant and antifibrinolytic activity during *P. aeruginosa *pneumosepsis, by a PAF-dependent mechanism. Besides its possible pathophysiological relevance, *in vitro *detection of e*xoU *gene in bacterial clinical isolates warrants investigation as a predictor of outcome of patients with *P. aeruginosa *pneumonia/sepsis and as a marker to guide treatment strategies.

## Background

One of the main features of sepsis is the infection-triggered activation of the inflammatory and coagulation systems. In the lungs, sepsis is associated with increased permeability of the alveolar/capillary barrier, neutrophil infiltration and extensive intra-alveolar fibrin deposition [1]. Changes in pulmonary fibrin turnover are also an important feature of severe pneumonia demanding mechanical ventilation and of acute respiratory distress syndrome (ARDS) [2]. Besides compromising the lung gas-exchange barrier, excessive alveolar clotting is harmful because surfactant components may be incorporated into fibrin with subsequent alveolar instability [3].

Pulmonary coagulopathy is the net result of increased local activation of the coagulation cascade, primarily driven by the tissue factor (TF) pathway, downregulation of natural coagulation inhibitors, and overexpression of plasminogen activator inhibitor-1 (PAI-1), a potent inhibitor of plasminogen activators that activate the fibrinolytic system [4]. Studies have shown that impaired fibrinolysis associated with increased PAI-1 levels in pulmonary edema fluid correlates with adverse outcomes of patients with ARDS [5].

*Pseudomonas aeruginosa *is a prominent agent of severe ventilator-associated pneumonia and sepsis [6]. A large body of evidence suggests that proteins delivered into host cells by the type III secretion system play a critical role in the pathogenesis of *P. aeruginosa-*induced sepsis and ensuing mortality [7]. Evidences are particularly compelling for ExoU, a toxin with phospholipase A_2 _(PLA_2_) activity [7], encoded by about 30% of clinical and environmental strains [8], and highly cytotoxic to a range of eukaryotic cells.

PLA_2 _belongs to a family of enzymes that cleaves the *sn-*2 ester bond of phospholipids from mammalian membranes into free unsaturated fatty acids, such as arachidonic acid, and lysophospholipids. Released arachidonic acid can be converted into eicosanoids that play a key role in the inflammatory process [9]. PLA_2 _is also crucial in the synthesis of platelet activating factor (PAF), a potent proinflammatory mediator [10]. Extensive clinical and investigational evidences indicate that dysregulated PAF signalling is involved and, in some cases, may be a critical determinant of ARDS and sepsis [11,12].

Regulatory mechanisms that control the PAF signalling system include PAF degradation by the enzyme PAF acetylhydrolases (PAF-AH) [10,13]. PAF-AH gene contains elements that confer responsiveness to inflammatory challenge. Accordingly, the expression of PAF-AH mRNA and protein can be upregulated by inflammatory stimuli, such as bacterial lipopolysaccharide [14], as well as by PAF itself [15].

We have shown that, due to its PLA_2 _activity, ExoU exhibits marked proinflammatory activity [16,17], induces TF mRNA and protein overexpression by airway epithelial cells [18] as well as vascular hyperpermeability, platelet activation and systemic thrombus formation in *P. aeruginosa*-infected mice [19]. However, the ability of ExoU to interfere with the alveolar coagulation/fibrinolysis balance has not yet been directly investigated.

In the present study we addressed the question of whether ExoU would upregulate PAI-1 expression by airway epithelial cells, and whether PAF would contribute to the generation of the ExoU-induced antifibrinolytic environment in mice airways. The current investigation was further motivated by studies reporting the PAF ability to activate PAI-1 gene expression by a PAF receptor (PAFR)-mediated mechanism [20].

## Methods

### Bacteria

*P. aeruginosa *PA103 strain and its *exoU *deficient mutant PA103Δ*exoU *[16] were grown in Luria-Bertani broth at 37°C for 14-16h, harvested by centrifugation and resuspended in sterile LPS-free saline or culture medium, as described [19].

### Mice infection

Female Swiss mice aged 8-12 weeks were infected intratracheally with 10^4 ^colony-forming units of PA103 or PA103Δ*exoU *in 50 μL of lipopolysaccharide -free saline, as described [19]. To assess the contribution of PAF in the procoagulant lung environment, 1 h before the bacterial instillation, mice were intraperitoneally inoculated with the PAFR inhibitor WEB 2086 (Biomol), at a final dose of 7.5 mg.kg^-1^. At 24 h post-infection, animals were euthanized by intraperitoneal injection of sodium pentobarbital, and their lungs were flushed with 1 mL of phosphate buffer saline pH 7.4 (PBS).

Recovered bronchoalveolar lavage fluids (BALF) were assessed for total cell counting using standard haemocytometer. Cytospin preparations were stained with Diff-Quick stain (Dade Diagnostics, Inc.) for leukocyte identification. In other sets of experiments, mice lungs were fixed in 10% formalin and embedded in paraffin. All animal experiments were performed in accordance with the guidelines of the Animal Ethics Research Committee of the State University of Rio de Janeiro (protocol # CEA/210/2007).

### BALF analysis

The following commercial kits were used: i) TNF-α and IL-6 were assayed using DuoSet immunoassay kits (R&D Systems, ref. DY 410 and DY 406, respectively); ii) TF-dependent procoagulant activity was assessed with the Actichrome TF activity assay kit (American Diagnostica Inc., ref. 846). Briefly, samples were mixed with factor VIIa and factor X and incubated at 37°C, allowing for the formation of the TF/factor VIIa complex (TF/FVIIa), and the factor X conversion to factor Xa. In the second stage of the reaction, the amount of factor Xa generated was measured by its ability to cleave Spectrozyme ^® ^Xa, a highly specific chromogenic substrate for factor Xa, added to the reaction solution. The cleaved substrate released a chromophore into the reaction solution. The solution absorbance was read at 405 nm and compared to those values obtained from a standard curve generated using known amounts of active TF; iii) Tissue Factor Pathway Inhibitor (TFPI) was measured with the IMUBIND Total TFPI ELISA kit (American Diagnostica Inc, ref. 849); iv) PAI-1 was assayed with the murine PAI-1 total antigen assay ELISA kit ELISA kit (Innovative Research, ref. MPAIKT-TOT); v) PAF-AH activity was measured using a PAF-AH Assay kit, (Cayman Chemical, ref. 760 901). This assay uses 2-thio PAF which serves as a substrate for PAF-AH. Upon hydrolysis of the acetyl thioester bond at the sn-2 position by PAF-AH, free thiols are detected using 5,5 - dithiobis(2-nitrobenzoic acid) supplied by the kit. The detection range of the assay is from 0.02 to 0.2 μmol/min/ml of PAF-AH activity which is equivalent to an absorbance increase of 0.01 to 0.1 per minute.

### Immunohistochemical localization of fibrin and PAI-1

Sections of mice lungs embedded in paraffin were deparaffinized in xylene, hydrated, treated with 10 mM citrate buffer (pH 6.0) at 95-98°C for 20 minutes for antigen retrieval, rinsed and treated with a 3% H_2_O_2 _in PBS for 10 minutes at room temperature, to inhibit the endogenous peroxidase activity. After rinsing in Tris buffer 0,05M (pH 7.4), sections were incubated overnight at 4°C with Tris buffer containing 1% bovine albumin and then treated with a monoclonal anti-human fibrin antibody (American Diagnostica Inc., ref. 350) at 1:600 (corresponding to 5,5 μgmL^-1^) or a polyclonal anti-mouse PAI-1 antibody (Innovative Research, ref. IASMPAI-GF) at 1:4000 (corresponding to 2,5 μg.mL^-1^). After 30 min at 4°C, the sections were rinsed and immunostained with a biotin free MACH 4™ Universal horseradish peroxidase-polymer detection kit (Biocare Medical), following the manufacturer's instructions. The reaction was developed using the diaminobenzidine chromogen kit (Biocare Medical). Sections were stained with hematoxylin and eosin and analyzed.

### Cell cultures

Airway epithelial cells from the A549 line and macrophages from the THP-1 line were used to further investigate the ExoU ability to induce PAI-1 synthesis. The concentrations of PAI-1 released by control and infected cells were normalized and reported as pg secreted by 10^5 ^cells.

A549 cells were seeded (3.0 × 10^4 ^cells/96 well) in F12 nutrient culture medium (Invitrogen Inc.) containing 10% foetal calf serum (FCS) and antibiotics, cultured for 48 h and infected with PA103 or PA103Δ*exoU *suspensions at a multiplicity of infection of about 100:1. Bacteria were then centrifuged onto cell monolayers (1000 × g for 10 min), to enhance close contact with host cells. Control cells were exposed to culture medium or, in some assays, were treated with PAF at 20 μM. mL^-1^. After 1 h at 37°C, control and infected cells were incubated with culture medium containing gentamicin at 300 μg.mL^-1^. After 24 h, cell supernatants were recovered for ulterior PAI-1 analysis. In some assays, cells were treated for 1 h prior to infection with a polyclonal anti-PAFR antibody (Cayman Chemical Co., ref. 160 602) at 2 μg.mL^-1^.

THP-1 cells were seeded (3.0 × 10^4 ^cells/96 well) in DMEM medium containing 10% FCS, 1 mM pyruvic acid, antibiotics and phorbol -12-myristate 13-acetate (PMA; Sigma-Aldrich) at 40 ng.mL^-1^, for the induction of monocyte-macrophage differentiation [21]. After 3 days, cells were rinsed, cultured in fresh medium without PMA for additional 3 days, infected for 1 h and treated with the gentamicin-containing culture medium, as described above. After 24 h, cell supernatants were recovered for ulterior analysis.

### Detection of PAI-1 mRNA by reverse transcriptase-polymerase chain reaction (RT-PCR)

A549 cells infected for 1 h and treated with the gentamicin-containing culture medium for additional 17 h were trypsinized and rinsed to extract total RNA using Rneasy kit (Qiagen). cDNA was synthesized from total RNA by reverse transcription using the SuperScript- III First-Strand synthesis system for RT-PCR (Invitrogen), and amplified under the following PCR conditions: denaturation at 94°C for 2 min; and 30 (PAI-1) or 23 (β-actin) cycles of denaturation at 94°C for 45 s, annealing at 52°C for 45 s and extension at 72°C for 45 s. An additional extension step of 5 min at 72°C was carried out after the last cycle. The primers used in the reactions were: 5'-CTG ACT TCA CGA GTC TTT CAG ACC-3' (PAI-1 sense); 5'-CCC ATG AAA AGG ACT GTT CCT GTG-3' (PAI-1 antisense); 5'-GTT GCT ATC CAG GCT GTG C-3' (β-actin sense) and 5'-GCA TCC TGT CGG CAA TGC-3' (β-actin antisense). PCR products were subjected to electrophoresis in a 2% agarose gel and densitometry was performed using LabImage software (Kaplan GmbH, Halle, Germany).

### Statistical analysis

Statistical significance was accepted at the P < 0.05 level by one-way ANOVA for multiple group analysis with a Bonferroni adjustment.

## Results

### ExoU induced a robust inflammatory response in mice airways

**Figure 1:**
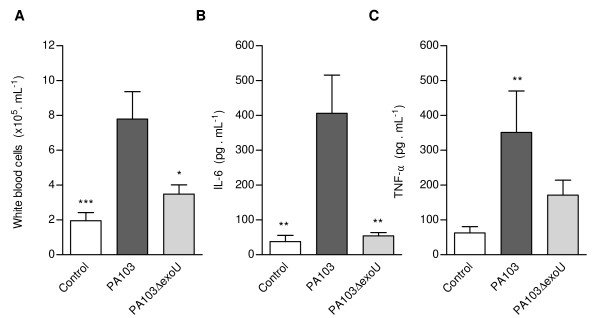
**ExoU-induced inflammatory environment in mice airways**. Concentrations of total white blood cells (A), IL-6 (B) and TNF-α (C) in BALF from PA103-infected mice (n = 24) were significantly higher than concentrations in BALF from control noninfected mice (n = 21) and from mice infected with the PA103 Δ*exoU *mutant (n = 21). * p < 0.05, ** p < 0.01 and *** p < 0,001 when mice infected with PA103 were compared with animals from the other two groups. Data represent mean values ± SE of the results obtained in three different assays.

BALF from mice infected with the ExoU-producing PA103 strain exhibited significantly increased concentrations of inflammatory cells (Figure 1A), IL-6 (Figure 1B) and TNF α (Figure 1C), confirming the proinflammatory activity of the toxin.

### ExoU also induced a procoagulant and antifibrinolytic environment in mice airways

**Figure 2:**
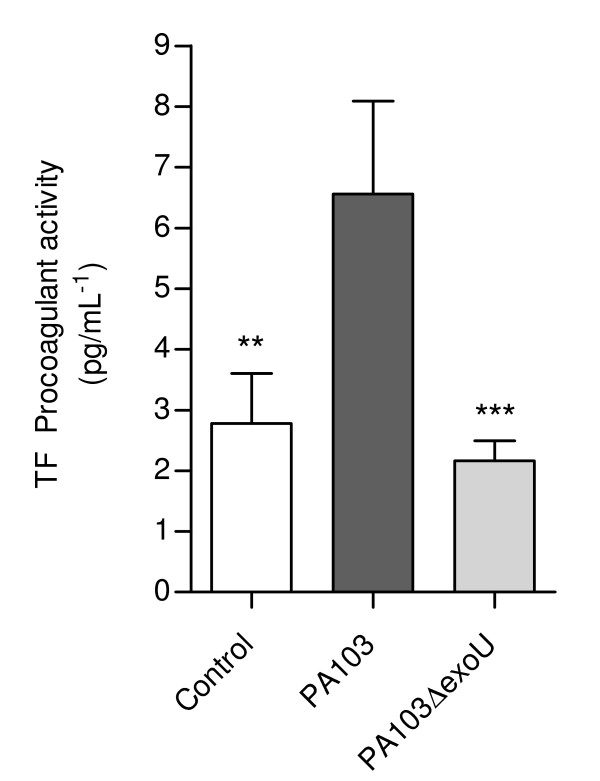
**ExoU-induced procoagulant activity in mice airways**. Tissue factor dependent procoagulant activity was significantly increased in BALF from PA103-infected mice. ** p < 0,01 and *** p < 0,001 when mice infected with PA103 (n = 12) were compared with those infected with the bacterial mutant (n = 10) or with control mice (n = 10). Data represent mean values ± SE of the results obtained in three different assays.

**Figure 3:**
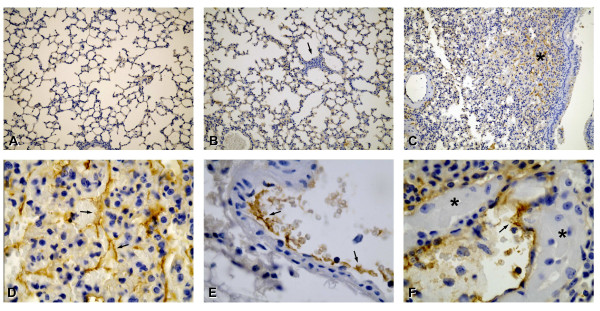
**Immunohistochemical localization of fibrin**. Lung sections from control mice (A) and PA103 Δ*exoU*-infected mice (B) exhibited minimal infiltration of inflammatory cells in interalveolar septum (arrow in B) and barely detectable fibrin staining. In sections from mice infected with the ExoU-producing bacteria, dark brown stained fibrin was detected all over the lung parenchyma (C; asterisk). Photomicrographs D-F exhibit higher magnifications of sections from PA103-infected mice. Arrows point to fibrin deposition in interalveolar septum (D) and inside blood vessels (E and F). Note in F, the huge edema in interalveolar space (asterisks). Original magnifications: A-C, × 20; D-F, × 100. Photomicrographs are representative of sections from seven animals from each group.

BALF from PA103-infected animals exhibited significantly elevated TF-dependent procoagulant activity (Figure 2). ExoU-induced local coagulopathy was confirmed by the immunohistochemical detection of widespread fibrin deposition in mice airspaces and lung microvasculature, that was not detected in animals infected with the ExoU-deficient mutant or in control mice (Figure 3).

**Figure 4:**
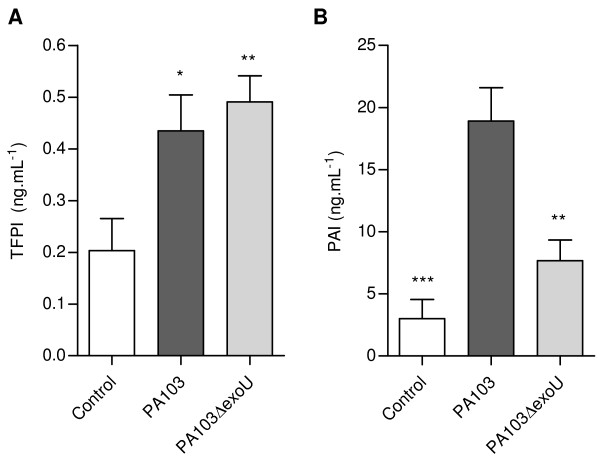
**ExoU-induced antifibrinolytic activity in mice airways**. In (A) it is shown that TFPI concentrations in BALF from mice infected with PA103 (n = 12) and PA103Δ*exoU *(n = 12) were significantly higher than concentrations in BALF from control mice (n = 13), but did not differ from each other. * p < 0.05 and ** p < 0.01 when infected mice were compared with control mice. In contrast, in (B) it is shown that PAI-1 concentration in BALF from mice infected with the ExoU-producing PA103 was significantly increased. ** p < 0.01 and *** p < 0,001 when mice infected with PA103 were compared with those infected with the bacterial mutant or with control mice. Data represent mean values ± SE of the results obtained in three different assays.

**Figure 5:**
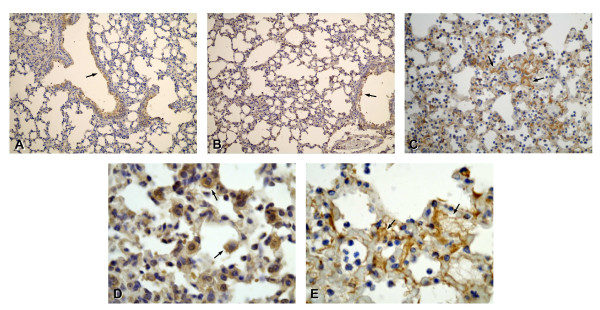
**Immunohistochemical localization of PAI-1**. In lung sections from control mice (A) and from mice infected with the bacterial mutant (B) PAI-1 staining was almost resctricted to the bronchiolar epithelium (arrows). Sections from mice infected with the ExoU-producing bacteria exhibited dark brown diaminobenzidine signals in intraalveolar fibrilar material reminiscent of hyaline membrane (arrows in C and E) and in type II pneumocytes (arrows in D). Original magnification: A-B, × 20; C, × 40; D and E, × 100. Photomicrographs are representative of sections from seven animals from each group.

Since the balances between the concentrations of TF and tissue factor pathway inhibitor (TFPI), as well as between local activation of coagulation and fibrinolysis are important determinants of alveolar fibrin deposition [4], we next assessed TFPI and PAI-1 levels in mice BALF. TFPI was significantly elevated in fluids from animals infected with both strains (Figure 4A). In contrast, PAI-1 concentration was significantly elevated only in BALF from PA103-infected mice (Figure 4B). ExoU-triggered overexpression of PAI-1 was confirmed by immunohistochemical analysis of mice lungs. In PA103-infected animals, PAI-1 was detected in lung parenchyma, bronchiolar epithelial cells and type II pneumocytes, as well as in a fibrillar material that accumulated in the alveolar compartment. In contrast, in lungs from control and PA103 Δ*exo*U-infected animals PAI-1 staining was restricted to the bronchiolar epithelium (Figure 5).

### ExoU modulated PAI-1 release by both airway epithelial cells and macrophages

**Figure 6:**
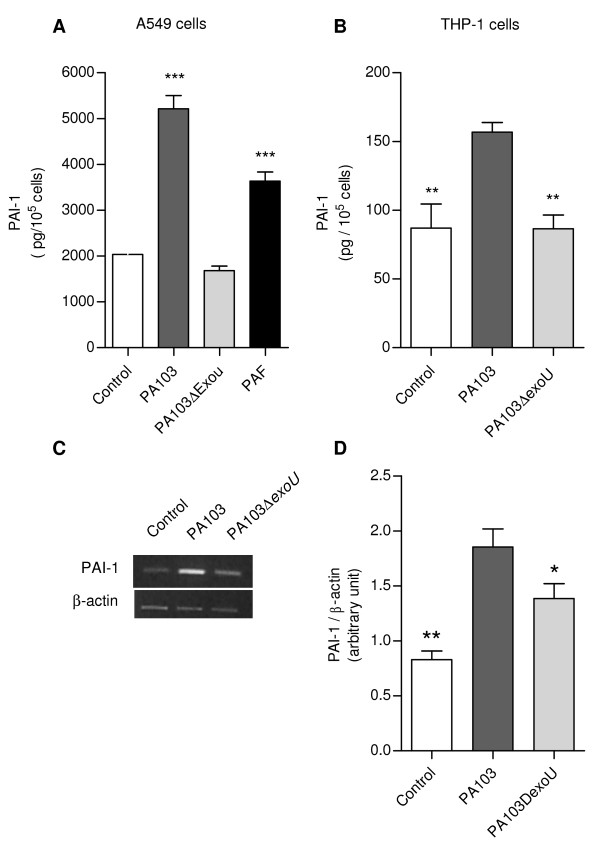
**Enhanced *in vitro *PAI-1 production by PA103-infected cells**. PAI-1 concentrations in supernatants from A549 epithelial respiratory cells (A) and THP-1 macrophages (B) infected with the ExoU-producing PA103 were significantly higher than concentrations detected in supernatants from control noninfected cells or from cells infected with the PA103 *exoU *mutant. Data represent mean values ± SE of the results obtained in three different assays carried out in quintuplicate. **p < 0.01 and *** p < 0,001 when data obtained from PA103-infected cells were compared with data from control cells and from PA104Δ*exoU*-infected cells, or when data obtained with PAF-treated cells were compared with those from control cells. In (C), a representative agarose gel shows the expression of PAI-1 and β-actin mRNA transcripts in control and infected A549 cells assessed by semiquantitative RT-PCR. In (D), mean ± SD values of the ratio of PAI-1 to β-actin transcript densities obtained in 2 RT-PCR assays carried out in duplicate. *, p < 0.05 and ** p < 0.01 when data obtained from PA103-infected cells were compared with those from controls and from PA103Δ*exoU*-infected cells.

PAI-1 concentrations in supernatants from both cell types infected with the ExoU-producing bacteria were significantly increased, but the concentrations detected in A549 cultures was about 30 fold higher than the concentrations in macrophage cultures (Figure 6A and 6B). PAI-1 release by A549 cells in response to ExoU stimulation was also significantly higher than the release induced by PAF (Figure 6A). Such modulation of PAI-1 release was likely dependent on increased transcription of the PAI-1 gene since in PA103-infected cells the level of PAI-1 mRNA was about 20% higher, in comparison with PA103Δ*exoU*-infected cells (Figure 6C).

### ExoU-induced PAI-1 overexpression was likely dependent on the PAF signaling system

**Figure 7:**
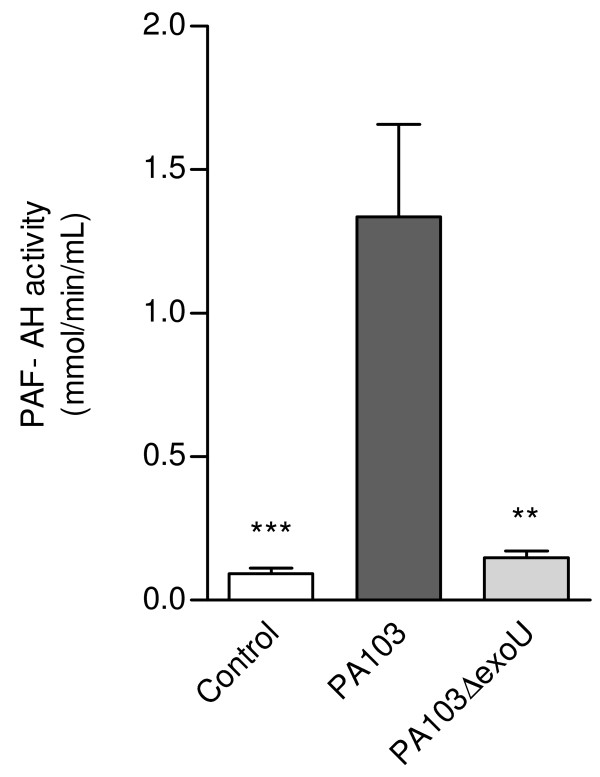
**BALF from PA103-infected mice exhibited increased PAF-AH activity**. PAF-AH activity in BALF from mice infected with the ExoU-producing bacteria (n = 13) was significantly higher than in BALF from control mice (n = 12) or from animals infected with the bacterial mutant (n = 13). ** p < 0,01 and *** p < 0,001 when mice infected with PA103 were compared with animals from the other two groups. Data represent mean values ± SE of the results obtained in three different assays.

**Figure 8:**
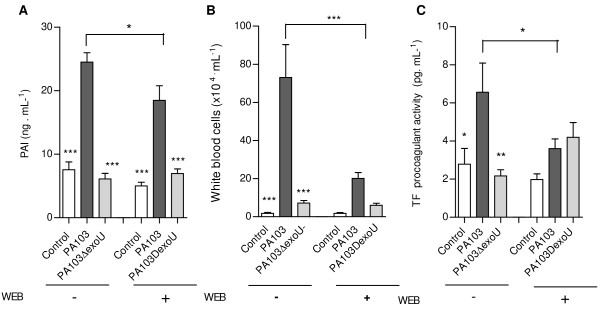
**Effects of mice treatment with the PAF antagonist WEB 2086**. Mice treatment with a PAF antagonist reduced significantly the concentrations of PAI-1 (A) and of inflammatory cells (B), as well as the procoagulant activity (C) detected in BALF from mice infected with the ExoU-producing PA103 bacteria. * p < 0,05, **, p < 0.01 and *** p < 0,001 when the results from PA103-infected mice pretreated with WEB (WEB +) were compared with those from untreated mice (WEB -), or when data obtained from PA103-infected mice were compared with those from controls or from PA103Δ*exoU*-infected animals. Each group contained at least 11 animals. Data represent mean values ± SE of the results obtained in three different assays.

To address the PAF contribution to PAI-1 release, we first investigated PAF-AH activity in mice BALF. The rationale for this approach was that since PAF was shown to stimulate the promoter activity of PAF-AH genes [15], and to increase the levels of PAF-AH mRNA in a dose-dependent manner [22], PAF-AH levels in host fluids are likely to reflect PAF concentrations. Since the enzyme activity was highly elevated in fluids from PA103-infected mice (Figure 7), we next investigated the effect of the PAF antagonist WEB-2086 on PAI-1 secretion. Figure 8A shows that WEB-2086 reduced significantly the PAI-1 levels in BALF from PA103-infected mice. We also observed a significant reduction of total leukocyte concentration (Figure 8B) and TF dependent procoagulant activity (Figure 8C) in fluids from mice infected with the ExoU-producing bacteria pretreated with the PAF antagonist.

**Figure 9:**
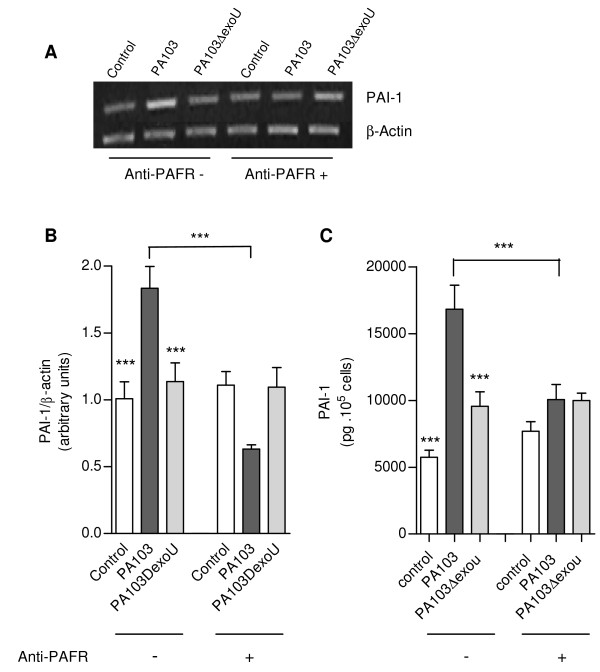
**Effects of anti-PAFR antibody on PAI-1 expression by PA103-infected cells**. In (A), a representative agarose gel shows the expression of PAI-1 and β-actin mRNA transcripts in control and infected A549 cells pretreated or not with the anti-PAFR antibody, assessed by semiquantitative RT-PCR. In (B), mean ± SD values of the ratio of PAI-1 to β-actin transcript densities obtained in 2 RT-PCR assays carried out in duplicate. In (C), mean values ± SE of PAI-1 concentration in cell supernatants from control and infected cultures, pretreated (anti-PAFR +) or not (anti-PAFR -) with the anti-PAFR antibody, obtained in three different assays carried out in quintuplicate. *** p < 0,001 when the results from PA103-infected cells pretreated with anti-PAFR antibody were compared with those from untreated cells or when data obtained from PA103-infected cells were compared with those from controls or from PA103Δ*exoU*-infected cells.

The role of PAF in PAI-1 overexpression was further demonstrated in *in vitro *assays. As shown in Figure 9, cell treatment with an anti-PAFR antibody prior to PA103 infection resulted in a significant reduction of both PAI-1 mRNA expression and PAI-1 concentration in cell culture supernatants. These results further support the contribution of the PAF signalling system in the ExoU-induced antifibrinolytic environment in mice airways.

## Discussion

This study demonstrated that ExoU enhances the following adverse events in the course of *P. aeruginosa *pneumosepsis: i) fibrin deposition in mice lung parenchyma; ii) alveolar TF-dependent procoagulant activity that did not depend on decreased TFPI production; iii) generation of an antifibrinolytic environment, secondary to increased PAI-1 production. The study also demonstrated the likely involvement of the PAF signalling system in ExoU-induced PAI-1 overproduction. Collectively, our results provide evidence of a novel mechanism that may contribute to the reported poor outcome of patients with severe infection by ExoU-producing *P. aeruginosa *[23].

Increased expression of TF has been widely reported to play a crucial role in initiating intra-alveolar coagulation and fibrin deposition in patients with ARDS [24,25]. The recently reported ability of ExoU to induce overexpression of TF mRNA and protein both *in vitro *and *in vivo *[18] may possibly be one of the key determinants of the currently described procoagulant activity in BALF from PA103-infected mice.

Mechanisms that regulate the coagulation pathway include TFPI, the sole endogenous TF inhibitor so far described. Studies have shown that the alveolar epithelium can regulate the release of TFPI into the air spaces in response to inflammatory stimuli [26]. Consistent with this idea, substantially increased TFPI concentrations were reported in BALF from patients at risk and with established ARDS [26]. Similarly, in our study, elevated TFPI levels were detected in BALF from mice infected with both *P. aeruginosa *strains but, in mice infected with the ExoU-producing bacteria, levels of alveolar TFPI were not sufficient to overcome the intense TF procoagulant activity induced by the bacterial toxin. A possible explanation for this insufficiency is the degradation of TFPI through the action of proteases found in the inflamed air spaces [27,28]. Indeed, the majority of TFPI detected in BALF from patients with ARDS was reported to be in inactive form [29].

A second mechanism that could contribute to the alveolar coagulopathy detected in PA103-infected mice is the impairment of the fibrinolytic activity, described in fluids from patients with ARDS [30], primarily attributable to increased PAI-1 expression [31]. The importance of upregulated PAI-1 levels as a marker of lung disease was recently substantiated by its recognition as a biomarker allowing the distinction between colonization and ventilator-associated pneumonia in mechanically ventilated patients [32].

In a recent investigation, high levels of PAI-1 detected in BALF from patients with *P. aeruginosa *ventilator-associated pneumonia/ARDS correlated with the production of type III ExoS and ExoU toxins by the infecting bacteria. Interestingly, patients infected with ExoU secreting strains had the highest PAI-1 levels [33]. This present study, which is the first to directly address the ability of ExoU to modulate PAI-1 expression, confirmed the existence of a positive relationship between ExoU expression and increased PAI-1 production *in vivo *and *in vitro*.

In this report, we also examined the role of PAF in ExoU-induced PAI-1 overexpression. The rationales were: i) cell intoxication with ExoU is likely to induce increased production of PAF because PAF synthesis by the remodelling pathway is initiated by the activity of PLA_2 _enzymes on membrane phospholipids [10]; ii) PAF is an inducer of PAI-1 gene expression [20].

To explore this possibility, it would have been ideal to demonstrate increased levels of PAF in BALF from PA103-infected mice. However, given the unstable nature of PAF, it is often problematic to measure this molecule *in vivo*. On the other hand, upregulation of PAF-AH expression is an important mechanism of PAF inactivation [14]. Since PAF itself is a potent inducer of PAF-AH release [15,22], PAF levels in mice BALF was indirectly estimated by the activity of PAF-AH, reported to be elevated in BALF from patients with ARDS [34]. Our finding that PAF-AH activity was greatly increased in fluids from PA103-infected mice was taken as an evidence of increased pulmonary PAF levels. Moreover, our results showing that a PAF antagonist reduced significantly the concentration of inflammatory cells and the procoagulant activity of BALF from PA103-infected mice were taken as a definitive evidence of increased levels of PAF induced by the ExoU-producing *P. aeruginosa *strain. Since PA103 and PA103Δ*exoU *only differ from each other in their ability to produce ExoU, we concluded that this toxin, and not LPS or another bacterial PAMP, accounted for increased PAF levels in mice airways. To our knowledge, this is the first report of a relationship between ExoU and PAF generation. Further studies are necessary to determine the role such PAF generation in the pathogenesis of *P. aeruginosa *infection.

Consistent with our hypothesis that PAF is involved in the control of PAI-1 production, treatment with the PAF antagonist significantly reduced PAI-1 levels in fluids from PA103-infected animals although the reduction was only partial. The biological effects of PAF are mediated by binding to a cell receptor [11] that is upregulated by inflammatory stimuli [35]. Indeed, ongoing immunocytochemical studies from our group have shown that PAFR is overexpressed in lung sections from PA103-infected mice (unpublished data). So, the inability to demonstrate a substantial inhibition of the ExoU-induced PAI-1 overproduction may have stemmed from difficulties in completely blocking the heavy expression of PAFR with the competitive inhibitor.

Since mice could not be treated with higher concentrations of the PAF antagonist, we further investigated our hypothesis by treating epithelial respiratory cells with an antibody against PAFR prior to infection. Strikingly, after cell treatment, the expression of PAI-1 mRNA by PA103-infected cells and the PAI-1 concentrations in cell culture supernatants were reduced to levels that did not differ from those detected in supernatants from control cells or from cells infected with the bacterial mutant.

## Conclusion

Our results support the hypothesis that, due to its procoagulant/antifibrinolytic activity, ExoU is implicated in the pathogenesis of severe sepsis and ARDS caused by *P. aeruginosa*.

Over the past years, studies have shown that detection of specific biomarkers in airspaces and plasma may favor the early detection of lung injury, the risk stratification of patient enrolled in clinical trials and the definition of specific therapies to individual patients [36]. Based on our results, we believe that *in vitro *detection of e*xoU *gene in clinical isolates deserves to be investigated as a predictor of patient outcome and a marker to guide treatment strategies.

## List of Abbreviations

ARDS: acute respiratory distress syndrome; BALF: bronchoalveolar lavage fluids; IL-6: interleukin-6; PAI-1: plasminogen activator inhibitor type 1; PAF: platelet-activating factor; PAFR: platelet-activating factor receptor; PAF-AH: platelet-activating factor acetylhydrolase; PBS: phosphate buffer saline; PLA_2_: phospholipase A_2_; RT-PCR: reverse transcriptase-polymerase chain reaction; TF: tissue factor; TFPI: Tissue Factor Pathway Inhibitor; TNF-α: tumor necrosis factor- α.

## Competing interests

The authors declare that they have no competing interests.

## Authors' contributions

GBM performed most of the assays. AVO performed immunohistochemical study analysis. AMS and CDM conceived and performed the molecular biology assays. JHRS contributed to the design of the study. MCP conceived and coordinated the study, participated in statistical analysis and wrote the manuscript. All authors read and approved the final manuscript.
